# Determinants of Adherence to Treatment in Hypertensive Patients of African Descent and the Role of Culturally Appropriate Education

**DOI:** 10.1371/journal.pone.0133560

**Published:** 2015-08-12

**Authors:** Jennita G. Meinema, Nynke van Dijk, Erik J. A. J. Beune, Debbie A. D. C. Jaarsma, Henk C. P. M. van Weert, Joke A. Haafkens

**Affiliations:** 1 Department of General Practice/Family Medicine, Academic Medical Center-University of Amsterdam, Amsterdam, the Netherlands; 2 Department of Social Medicine, Academic Medical Center-University of Amsterdam, Amsterdam, the Netherlands; 3 Department of Evidence-based medical education, University Medical Center of Groningen, Groningen, the Netherlands; Leibniz Institute for Prevention Research and Epidemiology (BIPS), GERMANY

## Abstract

**Background:**

In Western countries, better knowledge about patient-related determinants of treatment adherence (medication and lifestyle) is needed to improve treatment adherence and outcomes among hypertensive ethnic minority patients of African descent.

**Objective:**

To identify patient-related determinants of adherence to lifestyle and medication recommendations among hypertensive African Surinamese and Ghanaian patients with suboptimal treatment results (SBP≥140) living in the Netherlands and how culturally appropriate hypertension education (CAHE) influenced those determinants.

**Methods:**

This study analysed data of 139 patients who participated in the CAHE trial. Univariate logistic regression analysis was used to measure the association between patient-related determinants (medication self-efficacy, beliefs about medication and hypertension, social support, and satisfaction with care) and treatment adherence. We also tested whether CAHE influenced the determinants.

**Results:**

Medication self-efficacy and social support were associated with medication adherence at baseline. At six months, more medication self-efficacy and fewer concerns about medication use were associated with improved medication adherence. Self-efficacy was also associated with adherence to lifestyle recommendations at baseline. CAHE influenced patients’ illness perceptions by creating more understanding of hypertension, its chronic character, and more concerns about the associated risks.

**Conclusion:**

In this high-risk population, health care providers can support medication adherence by paying attention to patients’ medication self-efficacy, the concerns they may have about medication use and patients’ perceptions on hypertension. The CAHE intervention improved patients’ perception on hypertension.

## Introduction

Hypertension is a major risk factor for cardiovascular disease and especially stroke. [1] To reduce the risk of cardiovascular disease, hypertensive patients must be treated appropriately, either through lifestyle interventions alone or in combination with medication. [2] Treatment of hypertension fails when patient-related barriers towards treatment are not recognised. A better assessment and understanding of these barriers will allow optimal tailoring of interventions. [3]

In Western countries, hypertension is more common among ethnic minority groups of African descent than among whites. [4–6] Agyemang et al. reported that the prevalence of hypertension among native Dutch adults was lower than among adults of African-Surinamese and Ghanaian descent and that these ethnic minority groups had higher levels of hypertension awareness. [7] Among treated hypertensive patients, blood pressure control rates were significantly lower for the African Surinamese and the Ghanaians. [7–9] This demonstrates a need to address barriers to blood pressure control among African Surinamese and Ghanaian patients treated for hypertension. Poor adherence to treatment recommendations (medication and lifestyle) has been identified as a major modifiable cause of differences in blood pressure control rates in general [10, 11] and for those who belong to ethnic minority groups in particular [12, 13]. Knowledge of the relation between patient-related barriers and adherence could be used to enhance treatment-adherence among patients with hypertension. [11]

Since studies in several countries have shown that adherence to hypertension treatment recommendations is generally lower among disadvantaged populations, such as ethnic minority groups, it has been hypothesised that patients who belong to these groups may benefit from culturally tailored educational approaches. [14–17] Additionally, cultural factors have been shown to influence patients’ beliefs and practices concerning hypertension. [18–21] We have previously developed a protocol for culturally adapted hypertension education (CAHE) to support adherence to lifestyle and medication recommendations and blood pressure control in hypertensive patients of African descent. [22] The protocol combines the principles of motivational interviewing (5 A’s; i.e. Ask, Assess, Advise, Assist, and Arrange) [23] with those from Arthur Kleinmans’ model. Kleinmans’ model proposes that patient perceptions of disease and treatment can differ substantially from those of their healthcare providers. [24] The protocol focuses on asking essential questions to generate understanding by the patients about their health beliefs and their own condition in a multi-cultural environment. The main goal of the CAHE intervention was to improve patients’ health behaviour and, in turn, their blood pressure. Using a cluster randomised controlled trial we showed that CAHE leads to a higher reduction in diastolic blood pressure (DBP) (5.73 mmHg vs. 1.70 mmHg) and better adherence to lifestyle recommendations among African-Surinamese and Ghanaian patients with uncontrolled hypertension when compared to standard care. [22, 25]

Research based on models of health behaviour has provided evidence that adherence to treatment is a self-regulatory process and that patients’ ideas about their illness (*the consequence*, *timeline*, *personal-control*, *treatment-control and identity*) and medications are predictive of adherence to medication and lifestyle recommendations. [18–21, 26] In addition, several studies have shown that self-efficacy influences patients’ ability and skills to continue treatment. [27–29] Also satisfaction with care and social support have been identified as factors influencing adherence. [9, 18, 30–32] However, the pathway from patient education to adherence (and hypertension control) is complex and still poorly understood. This study aims at deepening our understanding of this pathway, in particular for the treatment of hypertension in a migrant population at high risk of cardiovascular disease. We report for the first time focus areas for the treatment of this population. To this end we performed a secondary analysis of the CAHE trial with the aim to identify 1) the (best) subset of determinants that predicted adherence to medication and lifestyle recommendations prior to the intervention, 2) how changes in those determinants were associated with adherence to medication and lifestyle recommendations after the intervention (at six months), and 3) we evaluated the role of CAHE by investigating to what extent this intervention had influenced any of the determinants of adherence, and particularly those that were associated with an improvement in treatment adherence.

## Methods

### Study design

This study analyses data that were collected among participants who completed the CAHE study. Full details of the design and methods of this cluster-randomised trial and the content of CAHE intervention have been published previously. [22, 25] Briefly, the study tested the effect of the CAHE intervention on blood pressure control and treatment adherence among Surinamese and Ghanaian patients with uncontrolled hypertension from four Dutch primary health care centres (PHCC) in Southeast Amsterdam. PHCCs allocated to the control group provided standard (guideline based) hypertension care to the study participants and PHCCs allocated to the intervention group provided usual care and three nurse-led individual CAHE sessions to study participants. [33] At the start of the intervention (t0) and after 6 months (t1), BP and other physiological measures were assessed and information on self-reported adherence to lifestyle and medication and patient-related determinants of adherence (perception on medication and hypertension, self-efficacy, social support, satisfaction with care) was collected through validated questionnaires. (*Table A in S1 File*) Socio-demographic data were obtained at the start of the intervention. The research assistants who performed the baseline and follow-up assessments received a specific training for the purpose of this study and were blinded to the study conditions. [25] All data was entered into SPSS Data Entry 4.0 (Ref: SPSS Inc, Chicago IL, USA).

**Fig 1:**
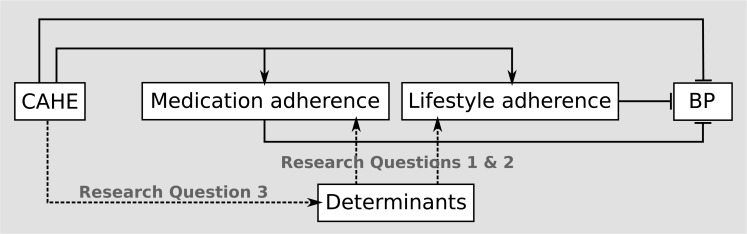
A schematic overview of the scope of this study. Fig 1 legend: A Schematic overview of the scope of this study and the study of Beune et al. [22] Beune et al. showed positive correlations (solid arrows) of the CAHE intervention with adherence to lifestyle recommendations. Additionally, CAHE was shown to lower diastolic blood pressure (blunt arrows). The dashed arrows indicate the research questions of this study. Research question 1 and 2 analyse which determinants are associated with adherence to medication and lifestyle recommendations. Research question 3 answers which determinants the CAHE intervention influences in order to increase adherence to medication and lifestyle recommendations.

For this paper we performed a stepwise analysis of 1) the association between background characteristics and patient-related determinants of adherence and self-reported adherence to medication and lifestyle recommendations at baseline, 2) the association between changes in patient-related determinants of adherence and changes in self-reported adherence of medication and lifestyle recommendations at six months and 3) the influence of the CAHE intervention on changes in patient-related determinants of adherence. (*Fig 1*)

### Variables and outcome measurements

#### Adherence to lifestyle and medication recommendations

*Self-reported adherence to medication recommendations* was assessed with the eight-item Morisky medication adherence scale (MMAS-8). [34] This scale has been well validated in several studies among African-American populations. [9, 35–37] The MMAS-8 asks patients to respond with ‘‘yes” or ‘‘no” to a set of 7 questions and to one 5-point Likert scale question. The score for full adherence is 8, with lower scores indicating a poorer level of adherence with a lower boundary of zero. In this study patients were described as non-adherent if they had an MMAS-8 score < 6 and as adherent if their score was ≥ 6. When patients had become more adherent between T0 and T1 or remained on the maximum score, they were categorised as ‘improved adherence’.

*Self-reported adherence to lifestyle recommendations* was assessed with a three-item scale derived from the Morisky scale. [38] This scale contains three questions: 1) Have you been advised by your PN/GP about smoking, nutrition, alcohol, weight control and/or physical activity (Yes/No)? 2) If yes, what advice was given? 3) To what extent did you follow this advice (range: never (1)–always (4))? On the basis of answers to these questions a composite score for adherence to lifestyle recommendations was computed (range: 1–4). Patients with scores 1 and 2 were categorised as non-adherent and those with scores 3 and 4 were categorised as adherent to lifestyle recommendations. When patients had become more adherent between T0 and T1 or remained on the maximum score, they were categorised as ‘improved adherence’. (*Results of a psychometric analysis of these outcome measures are shown in Tables D and E in S1 File*).

#### Background characteristics and patient-related determinants of adherence

Self-reported data on age, gender, educational level, financial status, duration of hypertension, and years living in the Netherlands were collected at the start of the study, to describe background characteristics of the study sample. (*Table B in S1 File*)

The following five measures were used to obtain information on potential patient-related determinants of adherence (*Table A in S1 File*):

*Perceptions of hypertension* were assessed with the Brief Illness Perceptions Questionnaire (*IPQ-brief*). The validity and the reliability of this questionnaire is well established The IPQ-brief asks patients to provide information about 8 dimensions of illness (hypertension) that can be answered on a 5-point Likert scale. Five dimensions are related to cognitive illness representations (the perceived consequence, timeline, personal-control, treatment-control and identity), two dimensions are related to emotional representations (respondents’ emotions and concerns about the illness) and one dimension is related to illness comprehensibility (coherence). [39] Separate scores for the 8 dimensions of illness perception and an overall score were computed. The scores represent the degree to which the illness is perceived as threatening (high scores) or benign (lower scores).

*Medication self-efficacy* was assessed with the Medication Adherence Self-Efficacy Scale (MASES-R). This scale shows good internal consistency, predictive validity, and convergent validity in African-American patients with hypertension. [40] The patients’ confidence in their ability to take medicine on time in various situations is measured with 13 questions, that can be answered on a 4-point Likert scale. [40] Scores were totalled and the mean score reflects the amount of medication self-efficacy, with higher scores designating higher self-efficacy.

*Beliefs about medication* were assessed with the ten-item Beliefs about Medicines Questionnaire (BMQ). [26] This instrument has good internal consistency and validity. [41, 42] The first scale (Specific-necessity) of this questionnaire assesses hypertensive patients’ beliefs about how necessary it is to take medications in order to improve/maintain their health. The second scale (Specific-concern) assesses respondents’ “concerns” about potential adverse consequences from taking their medications. The BMQ uses 5-point Likert questions ranging from 1 = “strongly disagree” to 5 = “strongly agree”. The respondents’ scores on each item were totalled. Higher scores indicate stronger beliefs about the necessity of taking medicines and concerns about adverse effects of medications.

The amount of *Social Support* patients experienced when dealing with their condition was assessed using the validated 12-item Duke Social Support Scale (DUSOCS). [43] As demonstrated by previous studies in hypertensive African-American patients, this scale has good validity and reliability [35] DUSOCS is divided into two subscales: social support from family (6-item scale) and social support from others than family (5-item scale). A higher total score reflects more social support. The open question that is used in DUCOCS was not taken into account in our analysis.

*Satisfaction with care* was measured using five items of the Consumer Quality Index-Diabetes [44] and five items of the Quote Migrant. [45] Similar Consumer Quality Index instruments have demonstrated good construct and discriminating validity and internal consistency in previous studies [46, 47] The Quote Migrant has been developed to measure the quality of care experienced by migrants groups in the Netherlands.[45] A higher score reflects a higher satisfaction with care.

### Statistical analysis

Data were analysed using SPSS version 19 (Ref: SPSS Inc, Chicago IL, USA). The distributions of continuous data were graphically assessed. Means and standard deviations were reported for normally distributed data and medians and quartiles for non-normal distributed data.

Univariate logistic regression analysis was conducted to assess the association between each determinant of adherence and each outcome variable (adherence to medication or lifestyle recommendations). Since the number of participants in the study was low, relative to the number of measured determinants, [25] a p-value of 0.3 in the univariate analysis was used as an upper threshold for inclusion in the multivariate logistic regression model. Using a stepwise backward (LR) method, variables were selected for the final model. Only determinants that were statistically significant (p < 0.05) contributors to the multivariate model were considered to be related to adherence. The same procedure was used to examine the association between changes in determinants of adherence and changes in adherence to medication and lifestyle recommendations.

To assess the influence of the CAHE intervention on the determinants of adherence we compared the change in the determinants between the intervention and the control group. Scores were compared using a chi-square test for categorical data and an independent t-test for normally distributed continuous data.

The study protocols were approved by the medical ethics committee of the Academic Medical Center in Amsterdam (protocol ID MEC 09/070 # 09.17.0725) and CCMO (NL27507.018.09). This trial is registered at the ISRCTN Register under registration number ISRCTN35675524 (http://www.controlled-trials.com/ISRCTN35675524). In accordance with the Declaration of Helsinki, written informed consent was obtained from all patients.

## Results

### Description of the population

Patients’ background characteristics at baseline are shown in Table B in S1 File. The average age of the patients was 53.9±9.8 years, and 47.5% were male. Table 1 shows that at the start of the intervention 58.0% and 64.7% of the patients were categorised as adherent to medication and lifestyle recommendations, respectively. Patients who were adherent to medication recommendations reported a longer duration of stay in the Netherlands (mean difference: 4.5 years (95% CI: 0.68–8.3) and less financial difficulties (mean difference: 0.17 (95% CI: 0.01–0.33) compared to non-adherent patients. All other baseline differences between the adherent and non-adherent group are marginal and summarised in Table B in S1 File. After six months diastolic, but not systolic blood pressure was positively correlated with adherence to medication and lifestyle recommendations. (*Table C in S1 File*)

**Table 1:** Patient scores on measures for treatment adherence and patient-related determinants of adherence taken at baseline and six months (N = 139).

Measures		Start of intervention	After six months	Range
**Adherence**	*Adherent to medication*, *n (%)*	65 (58.0%)	79 (65.3%)	
	*Adherent to lifestyle advice*, *n (%)*	75 (64.7%)	87 (75.5%)	
**Positive change in medication adherence**, ***n (%)*:**			63 (57.3%)	
**Positive change in lifestyle adherence, *n (%)*:**			55 (56.7%)	
**Social support family, *mean (±SD)***		39.5 (±28.3)	41.3 (±25.9)	0–100
**Social support others, *mean (±SD)***		25.9 (±23.2)	29.3 (±23.8)	0–100
**Illness perception**	*Illness perception overall score (IPQ-Brief)*, *mean (±SD)*	36.6 (±11.2)	34.3 (±10.2)	0–80
***Per item*:**	*Consequences*	5.0 (±3.1)	5.2 (±2.8)	0–10
	*Timeline*	6.0 (±3.2)	6.6 (±3.0)	0–10
	*Personal-control*	6.3 (±2.8)	7.4 (±2.1)	0–10
	*Treatment-control*	7.4 (±2.3)	8.1 (±1.7)	0–10
	*Identity*	4.1 (±3.1)	4.1 (±2.7)	0–10
	*Illness coherence*	7.2 (±2.9)	7.5 (±2.7)	0–10
	*Emotions*	5.0 (±3.4)	4.7 (±3.2)	0–10
	*Illness concerns*	7.2 (±3.0)	6.9 (±2.9)	0–10
**Medication self-efficacy, *mean (±SD)***		3.4 (±0.7)	3.5 (±0.6)	0–4
**Satisfaction with care**	*Satisfaction with healthcare—CQI*	18.4 (±2.4)	18.3 (±2.6)	6–20
	*Satisfaction with healthcare-Quote migrant*	12.2 (±5.9)	13.1 (±5.7)	0–20
**Beliefs about medication**	*Concerns about medication use–BMQ Concerns*	13.8 (±4.3)	13.6 (±4.4)	5–25
	*Necessity of medication use–BMQ Necessity*	16.4 (±5.0)	16.3 (±4.7)	5–25

SD = Standard deviation

### Treatment adherence and determinants of adherence

#### Association between treatment adherence at the start of the intervention and background characteristics and patient-related determinants of adherence

At baseline patients who were adherent to medication- and lifestyle recommendations reported higher medication self-efficacy as compared to non-adherent patients (*p*<0.001 and *p* = 0.026, resp.). Patients who were adherent to medications experienced less social support from non-family members as compared to non-adherent patients (p = 0.046). (*Table 2)* Adherence was not significantly associated with the other potential patient-related determinants.

**Table 2:** Association between adherence to medication and lifestyle recommendations and determinants at start of intervention.

***Determinants of medication adherence*** *^1^	***Exp*. *Β (95% Confidence Interval)***	***P-value***
Medication self-efficacy	7.62 *(3*.*00–19*.*35)*	*<0*.*001*
Social support—others	0.98 *(0*.*96–1*.*00)*	*0*.*046*
***Determinants of lifestyle adherence*** *^2^	***Exp*. *Β (95% Confidence Interval)***	***P-value***
Medication self-efficacy	2.16 *(1*.*10–4*.*27)*	*0*.*03*
Social support—others	1.02 (*1*.*00–1*.*04*)	*0*.*10*

* ^*1*^ *Multiple logistic regression analysis*, *backward LR*. *Model included variables*: *Medication self-efficacy*, *Illness perception overall score (IPQ-brief)*, *Social support–family*, *Social support–others*, *Satisfaction-healthcare*. *And not modifiable factors*: *age*, *financial status*, *duration of hypertension*, *and years in Netherland*.

* ^***2***^ *Multiple logistic regression analysis*, *backward LR*. *Model included variables*: *Medication self-efficacy*, *Social support—others*, *Social support–family*, *satisfaction healthcare*. *And non-modifiable factors*: *Age and educational level*.

#### Association between changes in patient-related determinants of adherence and changes in treatment adherence at six months

Table 3 shows that patients who showed an higher level of medication self-efficacy after six months were more likely to report an improvement in adherence to medication than those who did not (*p* = 0.031). Additionally, older patients were more likely to show an improvement in medication adherence than younger patients (p = 0.033). Patients who were more concerned about their medication use at baseline (BMQ-concern) were less likely to report an improvement in medication adherence. In this group the reported level of medication adherence remained low or decreased (p = 0.04). There were no significant associations between changes in determinants of adherence and changes in adherence to lifestyle recommendations.

**Table 3:** Associations between changes in adherence to medication and lifestyle recommendations and changes in determinants of adherence at six months.

***Determinants of medication adherence*** *^1^	***Exp*. *Β (95% Confidence Interval)***	***P-value***
*Change in* medication self-efficacy	2.64 *(1*.*09–6*.*39)*	*0*.*03*
*Change in* concerns about medication use	0.89 (*0*.*79–0*.*99*)	*0*.*04*
*Change in* illness perceptions	1.04 (*0*.*99–1*.*09*)	*0*.*10*
Age	1.05 *(1*.*00–1*.*11)*	*0*.*03*
***Determinants of lifestyle adherence*** *^2^	***Exp*. *Β (95% Confidence Interval)***	***P-value***
*Change in* medication self-efficacy	1.56 *(0*.*69–3*.*53)*	*0*.*24*

* ^*1*^ *Multiple logistic regression analysis*, *backward LR*. *Model included variables*: *the change in medication self-efficacy*, *Illness perception overall score (IPQ-brief)-*, *and beliefs about medication- (BMQ-concern)*. *And non-modifiable factors*: *age*, *educational level and duration of hypertension*.

* ^*2*^ *Multiple logistic regression analysis*, *backward LR*. *Model included variables*: *the change in medication self-efficacy*. *And non-modifiable factors*: *Age and educational level*.

#### Influence of the CAHE intervention on patient-related determinants of adherence.

Table 4 shows that patients who had received CAHE reported a better understanding of hypertension (IPQ-coherence (p = 0.028)), and more concerns about their hypertension than those in the control group (IPQ-illness concern (p = 0.033) and they better realized that hypertension is a chronic disease (p = 0.05)). However, there were no other statistically significant differences in scores on patient-related determinants of adherence between the two groups.

**Table 4:** The mean changes in patient-related determinants of adherence for patients allocated to CAHE (intervention) or usual care (control).

*Variable*	*Mean (SD)*		*P value*	*Mean difference (95% CI)*
	*Intervention*	*Control*		
*Change in* **social Support**	2.78 (18.66)	2.99 (22.25)	*0*.*95*	*-0*.*20 (-7*.*22–6*.*81)*
*Change in* **concerns about medication use**	-0.45 (3.94)	-0.33 (4.23)	*0*.*88*	*-0*.*11 (-1*.*68–1*.*45)*
*Change in* **necessity of medication use**	0.38 (4.68)	-0.43 (3.73)	*0*.*31*	*0*.*81 (-0*.*77–2*.*40)*
*Change in* **satisfaction healthcare**	0.12 (2.43)	-0.39 (2.61)	*0*.*25*	*0*.*51 (-0*.*35–1*.*36)*
*Change in* **satisfaction migrant**	1.20 (3.48)	0.55 (4.11)	*0*.*33*	*0*.*64 (-0*.*66–1*.*95)*
*Change in* **self-efficacy**	0.085 (0.68)	0.088 (0.44)	*0*.*98*	*-0*.*002 (-0*.*22–0*.*22)*
*Change in* **illness perceptions *overall***	-1.88 (9.13)	-3.18 (9.43)	*0*.*42*	*1*.*30 (-1*.*87–4*.*47)*
*Change in* ***consequence***	0.41 (2.80)	-0.15 (2.85)	*0*.*31*	*0*.*55 (-0*.*40–1*.*51)*
*Change in* ***timeline***	0.94 (2.51)	0.15 (2.85)	*0*.*05*	*0*.*79 (0*.*00–1*.59)*
*Change in* ***personal-control***	1.34 (3.07)	0.82 (2.77)	*0*.*25*	*0*.*51 (-0*.*48–1*.*51)*
*Change in* ***treatment-control***	0.97 (2.32)	0.54 (2.54)	*0*.*31*	*0*.*43(-0*.*40–1*.*25)*
*Change in* ***identity***	0.33 (2.93)	-0.44 (3.29)	*0*.*15*	*0*.*77 (-0*.*28–1*.*83)*
*Change in* ***coherence***	0.94 (3.12)	-0.22 (3.02)	*0*.*03*	*1*.*16 (0*.*12–2*.20)*
*Change in* ***emotions***	-0.06 (3.36)	-0.72 (3.10)	*0*.*23*	*0*.*66 (-0*.*43–1*.*76)*
*Change in* ***illness concern***	0.14 (2.74)	-0.87 (2.77)	*0*.*03*	*1*.*01 (0*.*08–1*.94)*

Comparison of the CAHE and usual care groups of their change in determinants at the start of the intervention and after 6 months

## Discussion and Conclusion

This study examined the pathway from patient education (CAHE) to adherence via patient-related determinants of adherence, among treated hypertensive patients of African descent from Dutch general practices with suboptimal treatment control (*Fig 1*). We found that medication self-efficacy and social support were the main determinants of adherence to lifestyle and medication recommendations at baseline. An increase in self-efficacy and a decrease in concern about medication use during the course of the clinical trial, led to an improvement in medication adherence. We found that the CAHE intervention did not significantly influence the aforementioned determinants of adherence. However, CAHE did improve patients’ illness perceptions, in terms of a better understanding of hypertension, more concerns about the associated risks and more awareness of the chronic character of hypertension.

### Comparison with other studies

This study demonstrates that patient’s medication self-efficacy positively influences adherence to antihypertensive medication. Similar results have been reported by studies on adherence [27, 48]: The more confident patients are that they will be able to take their medications in different situations, the more likely they are to be adherent. This is also consistent with findings from studies on other chronic diseases, such as rheumatoid arthritis, asthma, depression, [28, 32, 49, 50] and with studies on hypertension in African American patients. [27, 51, 52]

Our study suggests that adherent patients experienced a lower level of social support from people outside their families. Interestingly, this contrasts findings by Maguire et al., [53] who showed that adherent patients experience greater levels of social support. Although this last finding seems plausible, previous studies have shown that, especially in ethnic minority populations that were the focus of this study, talking about ones’ health problems to people outside the immediate family might be a taboo. Patients may fear that outsiders will view the fact that they have hypertension as an indication that their family suffers from financial problems or stress. [19, 54] This might explain the contrasting findings of Maguire et al. [53] and the present study.

In line with our finding that a lower level of concerns about medications is associated with improved adherence, a recent meta-analysis of 94 studies, that used the BMQ, also demonstrated that people with fewer concerns about their treatment show higher adherence to treatment. [55] This means it is important for health care providers to discuss cultural specific concerns about antihypertensives with patients from ethnic minority populations. Concerns about the side effects of medication, such as loss of sexual performance, or preference for traditional remedies, [18] may offer one possible explanation for the low rates of hypertension control found among African-Surinamese and Ghanaian men recently in the Netherlands. [56]

The study of Serour et al. [57] concluded that stress was mostly reported as the determinant for adherence to lifestyle recommendations. However, information on stress was not present in our data set. Since none of our examined determinants of adherence we examined in this study significantly explains the variation in adherence to lifestyle recommendations, stress (as reported by Serour et al. [57]) remains the only known determinant for lifestyle adherence.

### Study weaknesses and strengths

To our knowledge this is the first study that examines patient-related determinants of hypertension treatment adherence among ethnic minority patients of African descent with uncontrolled blood pressure outside the US. In the CAHE study self-report instruments for assessing adherence to medications and lifestyle recommendations have been used. The self-reported format to acquire data has general advantages and disadvantages. [58] To obtain a high level of reliability all determinants were tested using multiple questions with validated questionnaires *(Table A in S1 File).* Questions regarding lifestyle adherence were an exception since this scale was not validated. [25] However, the normal distributed outcomes ‘lifestyle adherence’ and ‘change in lifestyle adherence’ both provide Cronbach’s alpha that ensure enough confidence to use this instrument. (*Tables D and E in S1 File*)

The used Morisky scale for assessing lifestyle adherence is of a complex nature since it summarizes multiple items of lifestyle adherence (smoking, nutrition, alcohol, weight control and/or physical activity). Preferably the association of each item with the possible determinants would be tested individually on each item, which is unfeasible due to the limited population size.

Since this study is a secondary analysis, some measurements and instruments are not the best imaginable fit to obtain the answers to our research questions. Considering ‘medication adherence’ the actual medication intake would be a preferred measure, and also for ‘lifestyle adherence’ a more objective measurement would be preferred.

The self-efficacy reported for both lifestyle and mediation adherence is measured with the MASES-R. Since MASES-R is tailored to measure medication adherence, we can’t state that CAHE is not associated with lifestyle adherence self-efficacy. A possible explanation that CAHE did not affect medication-self efficacy is the limited room for improvement, as the level of medication self-efficacy was already very high at the start of the intervention (3.4 out of 4-point Likert scales).

The BMQ used to measure beliefs about medication was shown to be insufficient to capture cultural variations in medication beliefs. [55] As CAHE particularly targets culturally specific perceptions of medications the BMQ might be unsuitable to evaluate CAHE.

We have chosen the MMAS-8 for measuring medication adherence because the instrument has been well validated and had reported good predictive power. [9, 35–37] However it should be noted that some studies warn for overestimation of adherence by MMAS-8. [59]

Due to the relatively low numbers of participants we were forced to reduce the number of determinants that could be taken into consideration within our analysis. By excluding some of the determinants based on univariate relations, we also excluded interaction terms involving these determinants.

### Practice implications

The development of more effective methods for addressing treatment adherence among hypertensive patients of African descent is a priority for research and practice in Western countries. [7, 60] Our study suggest that interventions to support optimal adherence to prescribed medications are likely to be more successful in this population if health care professionals discuss patients’ concerns about medications and confidence in the ability to take medication (self-efficacy). While a previous study already has shown that CAHE can lead to greater adherence to lifestyle changes, [11, 18, 19] the present study suggests that this educational approach also leads to a better understanding of hypertension, and increase in concerns about the associated risks of hypertension and more awareness that hypertension can be controlled but not cured. Education should be targeted at the understanding that hypertension is not a disease in itself, but treatment is necessary to prevent events in the future, events that can be avoided. This is important since patients often stop taking medication or stop implementing lifestyle changes when their blood pressure is regulated as they believe hypertension is cured. These misconceptions about the nature and course of hypertension can negatively affect treatment adherence. [11, 18, 19] A possible option to tackle misconceptions and concerns about hypertension are shared medical appointments, which are shown to have a positive impact. [61, 62] As concerns about medication (and especially side-effects) lead to poor adherence and patients often do not mention (fear of) side-effects by themselves, doctors should discuss side-effects on a regular basis.

## Conclusion

Our study deepens the understanding of the pathway from patient education (CAHE) to adherence to lifestyle and medication recommendations for hypertension in ethnic minority patients of African descent with uncontrolled blood pressure. Firstly, more medication self-efficacy and less social support from people outside the family are associated with higher adherence to medication and lifestyle recommendations. Secondly, improvement in medication self-efficacy and decrease in concerns about medication is associated with increased medication adherence. Thirdly, the CAHE intervention appears to have a beneficial effect on patients’ illness perceptions, by contributing to a better understanding of hypertension and concerns about the associated risks, but not on patients’ medication self-efficacy or their concerns about medication.

## Supporting Information

S1 File Table A: Instruments for measuring patient-related determinants. Table B: Patient Characteristics at the start of the intervention (N = 139). Table C: Association between blood pressure and adherence. Table D: Extra information about outcome measure ‘Lifestyle adherence’. Table E: Extra information about outcome measure ‘Change in Lifestyle adherence’. (DOC)

S1 Text Dutch abstract. (DOC)
