# Supplementary material for: Determinants of Adherence to Treatment in Hypertensive Patients of African Descent and the Role of Culturally Appropriate Education
Source: PLoS One. 2015 Aug 12;10(8):e0133560. doi: 10.1371/journal.pone.0133560 (PMC4534399; doi:10.1371/journal.pone.0133560)
Supplement: S1 File — (DOC) [file pone.0133560.s001.doc]

**Supporting information file S2**

Tables of values behind the means, standard deviations and other measures reported

- S2A Table: Instruments for measuring patient-related determinants
- S2B Table: Patient Characteristics at the start of the intervention (N=139)
- S2C Table: Association between blood pressure and adherence
- S2D Tables: extra information about outcome measure ‘adherence to lifestyle recommendations’ at T0 and T1.
  - Chart S2A and S2B: Mean scores of ‘adherence to lifestyle recommendations’ at T0 and T1.
- S2E Tables: extra information about outcome measure ‘Change in adherence to lifestyle recommendations’
  - Chart S2C: Scores of ‘change in adherence to lifestyle recommendations’.
- S2F Table: extra information about outcome measure ‘Change in adherence to medication use’

| **Table S2A. Instruments for measuring patient-related determinants** | | | |
| --- | --- | --- | --- |
| **Measures** | **Instrument name** | **Items (range)** |  |
| **Adherence** | **Name of the scale [literature]** | ***N* items - *Range*** | **Interpretation** |
| **Self-reported medication adherence** | Morisky medication adherence scale (MMAS-8) | 8 (yes (0) or no (1))  *Range 0-8* | **- not adherent (<6)**  **- adherent (6 to 8)** |
| **Adherence to Lifestyle recommendations** | Questions derived from Morisky | 3 (never (1) – always (4))  *Range 0 - 4* | **- not adherent (≤ 2.5)**  **- adherent (>2.5)** |
| ***Determinants*** | | | |
| **Social Support** | DUSOCS | 12 (never (0) – very often (2))  *Range 0 - 100* | **Sum-score (0-100) reflects amount of social support** |
|  |  |  |  |
|  |  |  |  |
| **Medication Self -efficacy** | MASES-R11 | 13 (not at all sure (1) – extremely sure (4))  *Range 0 - 4* | **The mean of the total score reflects amount of medication self-efficacy** |
| **Illness perception**  **Brief IPQ (overall)**  *Consequence*  *Timeline*  *Personal-control*  *Treatment-control*  *Identity*  *Coherence*  *Emotions*  *Illness concern* | IPQ-brief | 8 (never (1) – always (10))  *Range 0 - 80* | **Higher overall score reflects amount of patients view on hypertension as more threatening** |
| **Beliefs about medication**  **BMQ – concern**  **BMQ - necessity** | BMQ | 2*5 (strongly agree (1) – strongly disagree(2))  *Range 5-25* | **Mean of the total score reflects amount of perceptions and beliefs on medication.** |
| **Satisfaction with care**  **Satisfaction-healthcare**  **Satisfaction-migrant** | CQI-diabets and Quote Migrant | 2*5 (never (1) – always (4))  *Range 6-20*  *Range 0-20* | **Sum-scoring of all items reflects amount of satisfaction with care** |

| **Table S2B. Patient Characteristics at the start of the intervention (N=139)** | | | | | | | | | | |
| --- | --- | --- | --- | --- | --- | --- | --- | --- | --- | --- |
| **Characteristics** | **Total group** | | **Adherent group for medication** | | | **Non-adherent group for medication** | | **Adherent group for lifestyle** | **Non-adherent group for lifestyle** | |
| **Age, years *mean (± SD)*** | | ***53.9 (±9.8)*** | | ***54.7 (9.6)*** | ***52.2 (9.5)*** | | ***54.1 (8.5)*** | | | ***51.7 (9.6)*** |
| **Sex *n* *(% male)*** | | ***66 (47.5%)*** | | ***22 (46.8%)*** | ***34 (52.3%)*** | | ***39 (52%)*** | | | ***21 (51.2%)*** |
| **Educational level *n (%)***  ***Low***  ***Middle***  ***High*** | | ***70 (50.4%)***  ***42 (32.2%)***  ***27 (19.4%)*** | | ***27 (41.5%)***  ***22 (33.8%)***  ***16 (24.6%)*** | ***22 (46.8%)***  ***17 (36.2%)***  ***8 (17%)*** | | ***35 (46.7%)***  ***25 (33.3%)***  ***15 (20.0%)*** | | | ***25 (61.0%)***  ***9 (22.0%)***  ***7 (17.1%)*** |
| **Financial status*, n (%)***  ***Can’t get by*** | | ***32 (23.4%)*** | | ***10 (15.4%)*** | ***15 (31.9%)*** | | ***19 (25.3%)*** | | | ***11 (26.8%)*** |
| **Years in the NL *(mean ±SD)*** | | ***24.9 (±10.0)*** | | ***26.6 (10.6)*** | ***22.1 (8.9)*** | | ***24.9 (10.4)*** | | | ***24.1 (8.6)*** |
| **Duration of high blood pressure *(mean ±SD)*** | | **9.12 (±8.83)** | | **10.0(9.2)** | **9.7 (7.9)** | | **8.0 (7.7)** | | | **9.0 (7.7)** |

| **Table S2C. Association between blood pressure and adherence** | | | | |
| --- | --- | --- | --- | --- |
| **Systolic blood pressure** | | | **Diastolic blood pressure T0** | |
|  | *Mean diff (sd)* | ***P value*** | *Mean diff (sd)* | ***P value*** |
| **Medication adherence t0** | **2.2 (2.0)** | **0.28** | **1.6 (1.6)** | **0.34** |
| **Lifestyle adherence t0** | **3.1 (2.3)** | **0.18** | **1.8 (1.8)** | **0.32** |
| **Medication adherence t1** | **3.3 (2.8)** | **0.24** | **4.0 (1.9)** | **0.04** |
| **Lifestyle adherence t1** | **5.5 (3.1)** | **0.08** | **6.7 (2.1)** | **0.002** |
| **Medication adherence: Positive change or adherence remain on the maximum score** | **0.3 (2.7)** | **0.91** | **0.1 (1.6)** | **0.96** |
| **Lifestyle adherenc: Positive change or adherence remain on the maximum score** | **4.3 (3.3)** | **0.20** | **6.2 (2.3)** | **0.009** |

*The general trend is that the adherent patients have lower blood pressure than the non-adherent patients.*

| **Table S2D. Characteristics of Lifestyle adherence (T0 and T1)** | | | | | |
| --- | --- | --- | --- | --- | --- |
| **Lifestyle adherence T0** | ***N*** | **Cumm. percentage** | **Mean** | **median** | **Cronbach’s Alpha** |
| Non-adherence (score =< 2.5) | 41 | 35.3 % | 2.8 | 2.8 | 0.93 |
| Adherence (score > 2.5) | 75 | 64.7 % |
| **Lifestyle adherence T1** |  |  |  |  |  |
| Non-adherence (score =< 2.5) | 28 | 24.3 % | 2.9 | 3.0 | 0.99 |
| adherence(score > 2.5) | 87 | 75.7% |

*The outcomes ‘lifestyle adherence’ and ‘change in lifestyle adherence’ approach a normal distribution and therefore floor- and ceiling effects are excluded.* *The Cronbach’s alpha of the outcome measure ‘lifestyle adherence at T0’ is: 0.93 and for ‘lifestyle adherence at T1 is: 0.99. The Cronbach’s alpha of the outcome measure ‘change in adherence’ is: 0.64. We used a 0.7 threshold value for the Cronbach's alpha, indicating that the alpha of adherence at T0 and T1 is good and for change in adherence near adequate.*

**Chart S2A: Adherence to lifestyle recommendations (T0)**

**Chart S2A: Adherence to lifestyle recommendations (T0)**


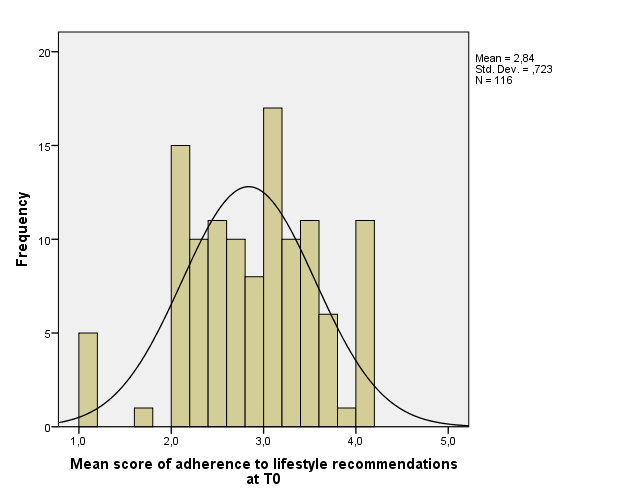


**Chart S2B: Adherence to lifestyle recommendations (T1)**


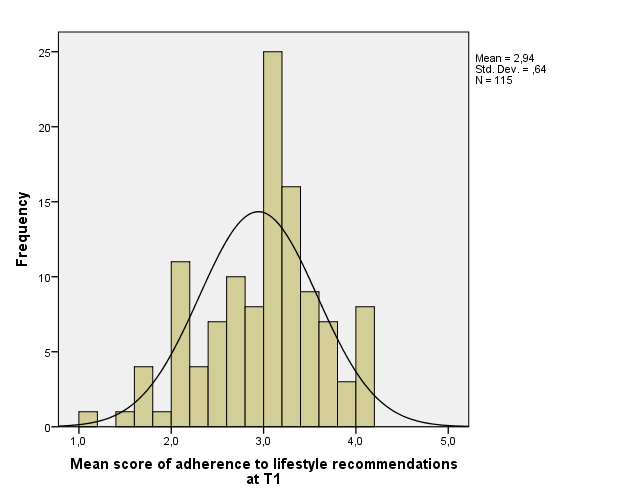


| **Table S2E. Characteristics of Change in lifestyle adherence** | | | | | |
| --- | --- | --- | --- | --- | --- |
| **Lifestyle adherence T1-T0** | ***N*** | **Cumm. percentage** | **Mean** | **Median** | **Cronbach’s Alpha** |
| Negative or no change in adherence | 42 | 43.3 % | 0.11 | 0.13 | 0.64 |
| Positive change or adherence remain on the maximum score | 55 | 56.7 % |  |  |
|  |  |  |  |  |  |


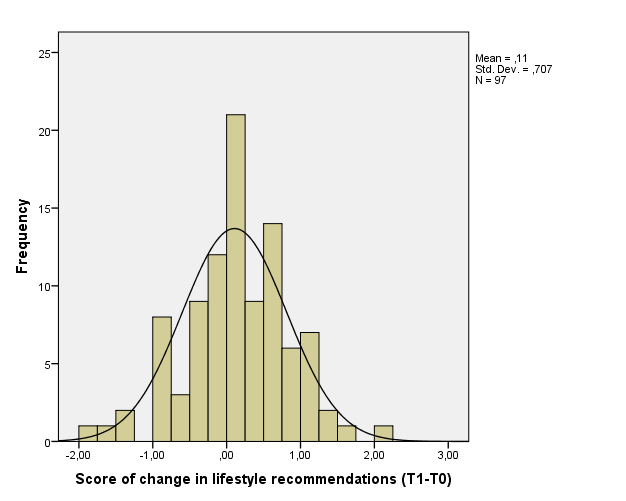
**Chart S2C: Change in adherence to lifestyle recommendations (T1-T0)**

| **Table S2F. Characteristics of Change in medication adherence** | | | |
| --- | --- | --- | --- |
| **Lifestyle adherence T1-T0** | **number** | **Cumm. percentage** | **Cronbach’s Alpha** |
| Negative or no change in adherence | 47 | 42.7 % | 0.71 |
| Positive change or adherence remain on the maximum score | 63 | 57.3 % |
|  |  |  |  |
