# Supplementary material for: Determinants of Adherence to Treatment in Hypertensive Patients of African Descent and the Role of Culturally Appropriate Education
Source: PLoS One. 2015 Aug 12;10(8):e0133560. doi: 10.1371/journal.pone.0133560 (PMC4534399; doi:10.1371/journal.pone.0133560)
Supplement: S1 Text — (DOC) [file pone.0133560.s002.doc]

**Supporting information file S1**

**Dutch Abstract**

Achtergrond: In westerse landen is meer en betere kennis nodig over patiënt-gerelateerde determinanten van het al- dan niet therapietrouw (medicatie en leefstijl) zijn om de naleving van behandeling en de resultaten bij hypertensieve patiënten van etnische minderheidsgroepen te verbeteren.

Doelstelling: Identificatie van patiënt-gerelateerde determinanten van het al- dan niet therapietrouw zijn onder Afrikaanse Surinaamse en Ghanese patiënten met een ongecontroleerde hypertensie (SBP>= 140), wonend in Nederland en hoe een cultureel sensitieve voorlichting (CAHE) deze determinanten beïnvloeden.

Methode: Deze studie analyseert gegevens van 139 patiënten die deelnamen aan de interventie CAHE. Een univariate logistische regressie analyse werd gebruikt om de associatie tussen patiënt-gerelateerde determinanten (medicatie self-efficacy, overtuigingen over medicatie en hypertensie, sociale steun en tevredenheid met zorg) en therapietrouw te bepalen. Met een onafhankelijke t-toets is gekeken of CAHE deze determinanten mogelijk beïnvloed.

Resultaten: Medicatie self-efficacy en sociale steun waren geassocieerd met het getrouw innemen van medicijnen voorafgaand aan de interventie. Na zes maanden waren meer medicatie self-effecacy en minder bezorgdheid over medicijngebruik geassocieerd met meer therapietrouw voor medicatie gebruik. Self-efficacy was eveneens geassocieerd met therapietrouw aan leefstijl adviezen. De interventie CAHE beïnvloed ziekte percepties van de patiënt: patiënten hadden een beter begrip van hypertensie en maakten zich meer zorgen om de ziekte. Tevens besefte meer patiënten uit de CAHE-groep dat de ziekte chronisch is en dus hun hele leven zal duren.

Conclusie: Medicatie Self-effacacy en bezorgdheid over medicijngebruik waren de belangrijkste patiënt-gerelateerde determinanten voor meer therapietrouw. CAHE beïnvloed deze determinanten echter niet. In deze bevolkingsgroep zouden zorgverleners therapietrouw kunnen beïnvloeden door aandacht te besteden aan self-efficacy, bezorgdheid over medicijngebruik en de percepties die patiënten hebben ten aanzien van de ziekte.
